# Supplementary figures and images for: Using an antimalarial in mosquitoes overcomes Anopheles and Plasmodium resistance to malaria control strategies
Source: PLoS Pathog. 2022 Jun 10;18(6):e1010609. doi: 10.1371/journal.ppat.1010609 (PMC9223321; doi:10.1371/journal.ppat.1010609)

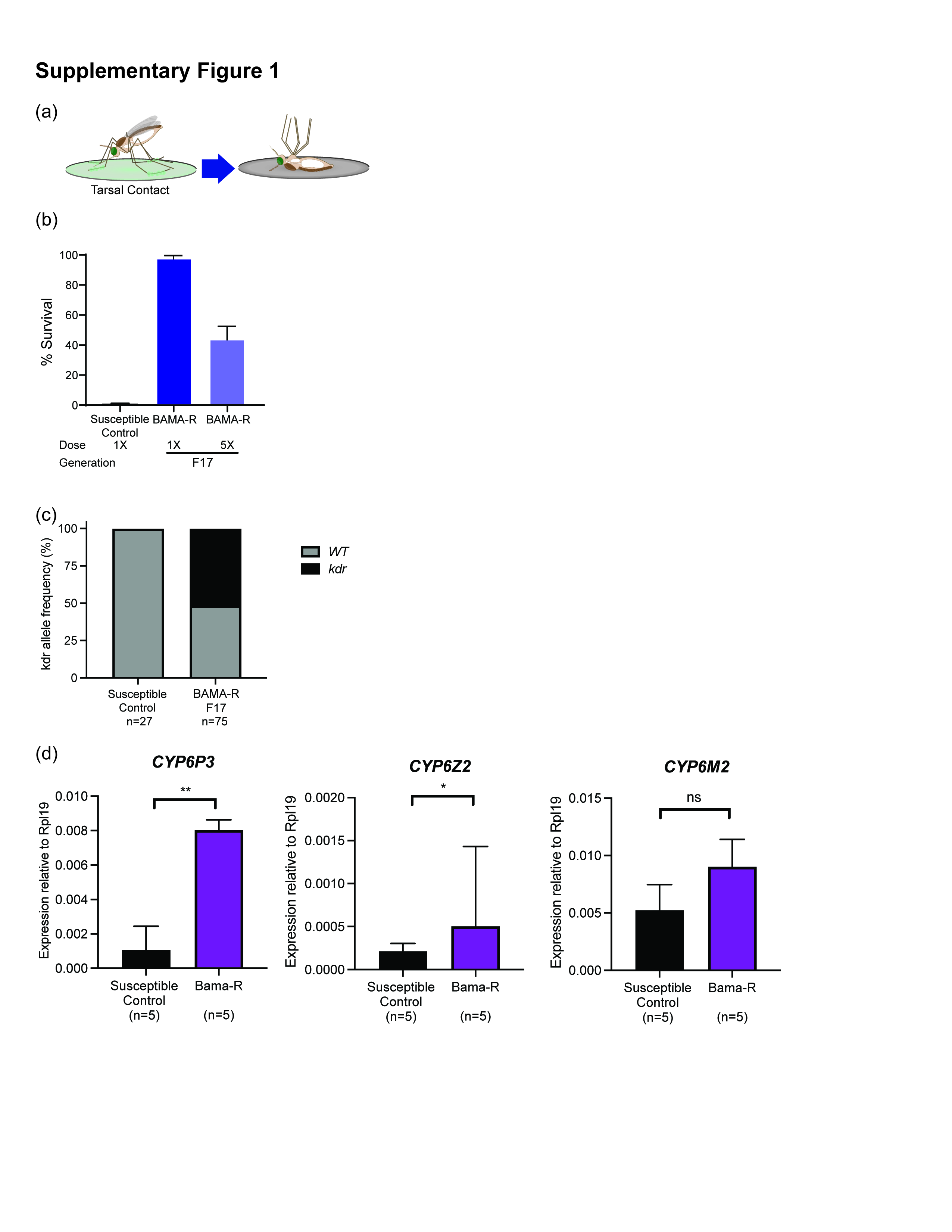

Supplement: S1 Fig — (a) Experimental scheme. (b) G3 females were 0% resistant at the DC, while 97% of exposed Bama-R females survived at the same dose, and 43% survived exposure to 5xDC, indicating extreme permethrin resistance. Mean survival ± SEM from 3 replicates is indicated. (c) Bama-R mosquitoes are segregating for the kdr allele conferring target site resistance to pyrethroids. Allele frequency at generation F17 was 51.7% (n = 75), indicating that the observed resistance phenotype is the result of additional modalities. (d) qPCR analysis shows that key cytochrome P450 genes associated with metabolic insecticide resistance are constitutively upregulated in Bama-R females compared to a susceptible control (G3). Median expression level normalized to the Anopheles housekeeping gene rpl19 are shown, error bars represent the interquartile range (IQR). Expression levels for Cyp6P3 and Cyp6Z2 were significantly elevated compared to a phenotypically susceptible control (Cyp6P3, Mann-Whitney, n = 10, df = 1, U = 0, p = 0.0079; Cyp6Z2, Mann-Whitney, n = 10, df = 1, U = 2, p = 0.0317) while Cyp6M2 was not significantly upregulated. Statistical significance is indicated where relevant as follows: ns = not significant, * = p<0.05, ** = p<0.01, *** = p<0.001, **** = p<0.0001. (TIF) [file ppat.1010609.s001.tif]

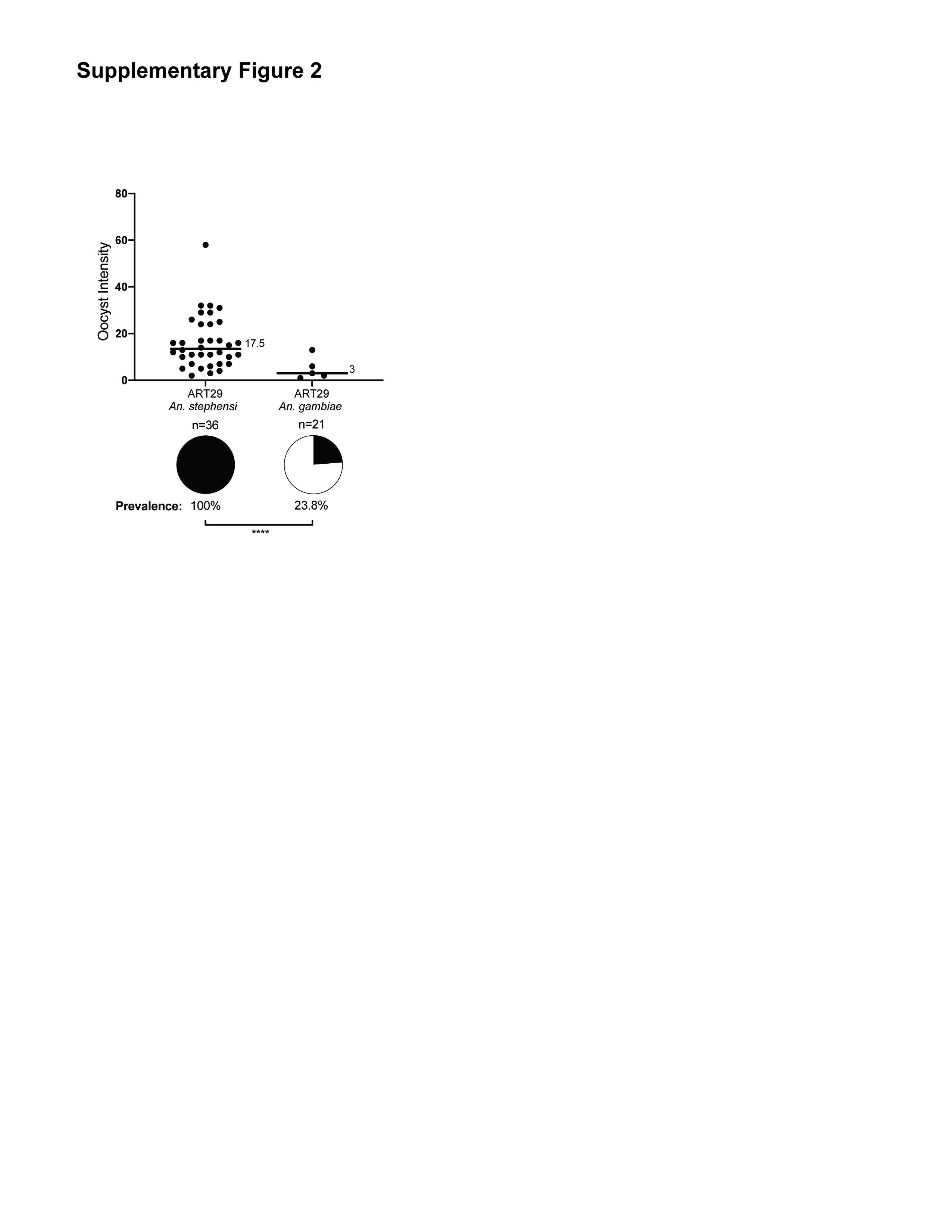

Supplement: S2 Fig — Female G3 (An. gambiae) and Anst (An. stephensi) were provided with a blood meal containing mature ART29 gametocytes. Outcome of infection was determined at 7 d pIBM by oocyst count. While ART29 exhibited poor infectivity in An. gambiae (23.8% infection prevalence), they established robust infections in An. stephensi (100% infection prevalence). (TIF) [file ppat.1010609.s002.tif]

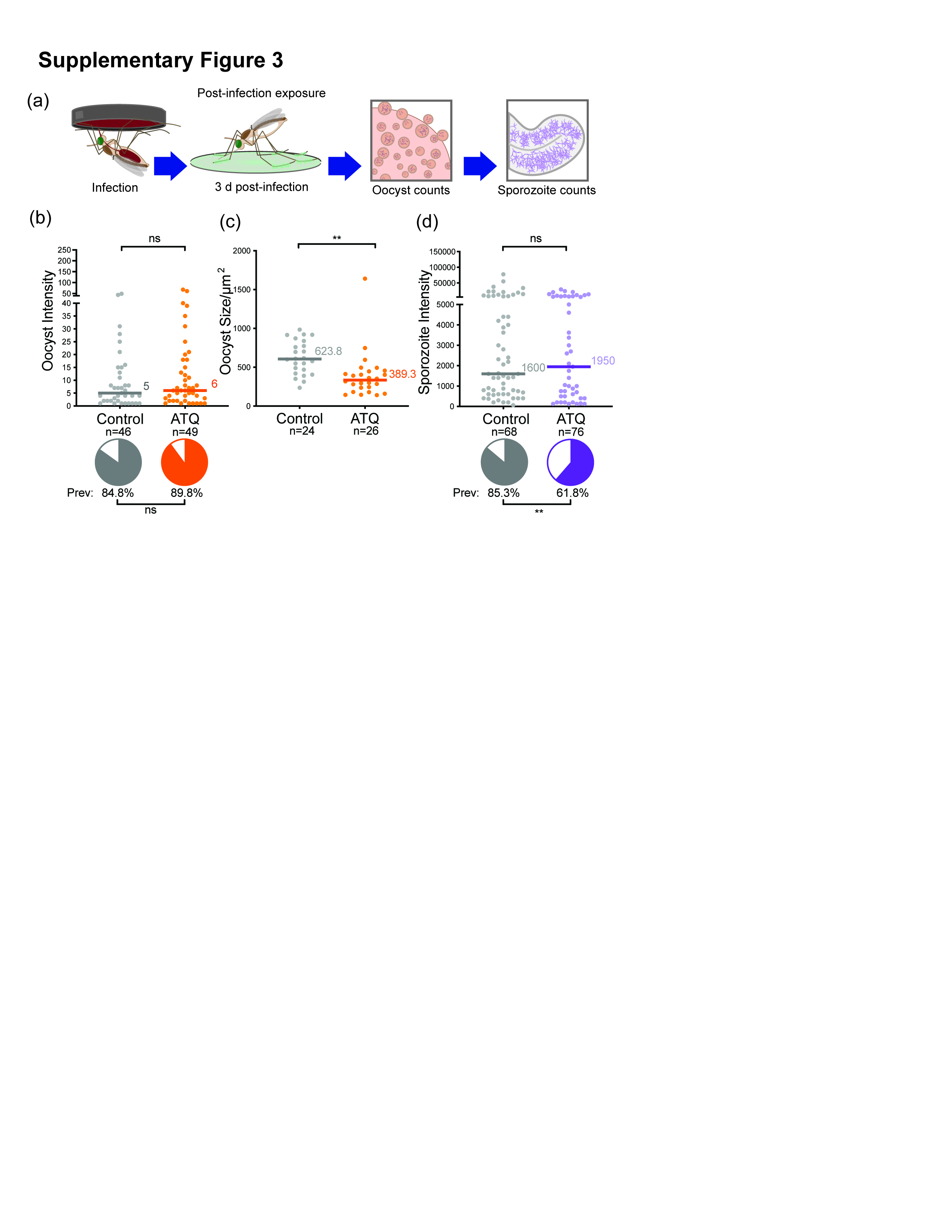

Supplement: S3 Fig — (a) Experimental scheme. (b) There was no effect of 3 d pIBM ATQ exposure on either prevalence (indicated by pie charts) or intensity (indicated by points) of infection determined at 10 d pIBM. Prevalence: Chi2, n = 95, df = 1, Χ2 = 0.540, p = 0.4623, intensity: Mann-Whitney, n = 80, df = 1, U = 717.5, p = 0.4530. (c) ATQ exposure at 3 d pIBM significantly reduced the median cross-sectional area of oocysts at 7 d pIBM relative to control (Chi2, n = 50, df = 1, U = 110, p<0.0001). (d) The prevalence, but not the median intensity of P. falciparum sporozoites in mosquito salivary glands was significantly reduced in mosquitoes exposed to ATQ at 3 d pIBM (Chi2, n = 144, df = 1, Χ2 = 9.995, p = 0.0016). Median lines and values are indicated, “n” indicates the number of independent samples. To isolate Oocyst/Sporozoite Prevalence and Oocyst/Sporozoite Intensity, midgut samples with zero oocysts have been excluded from intensity analysis. Statistical significance is indicated where relevant as follows: ns = not significant, * = p<0.05, ** = p<0.01, *** = p<0.001, **** = p<0.0001. (TIF) [file ppat.1010609.s003.tif]

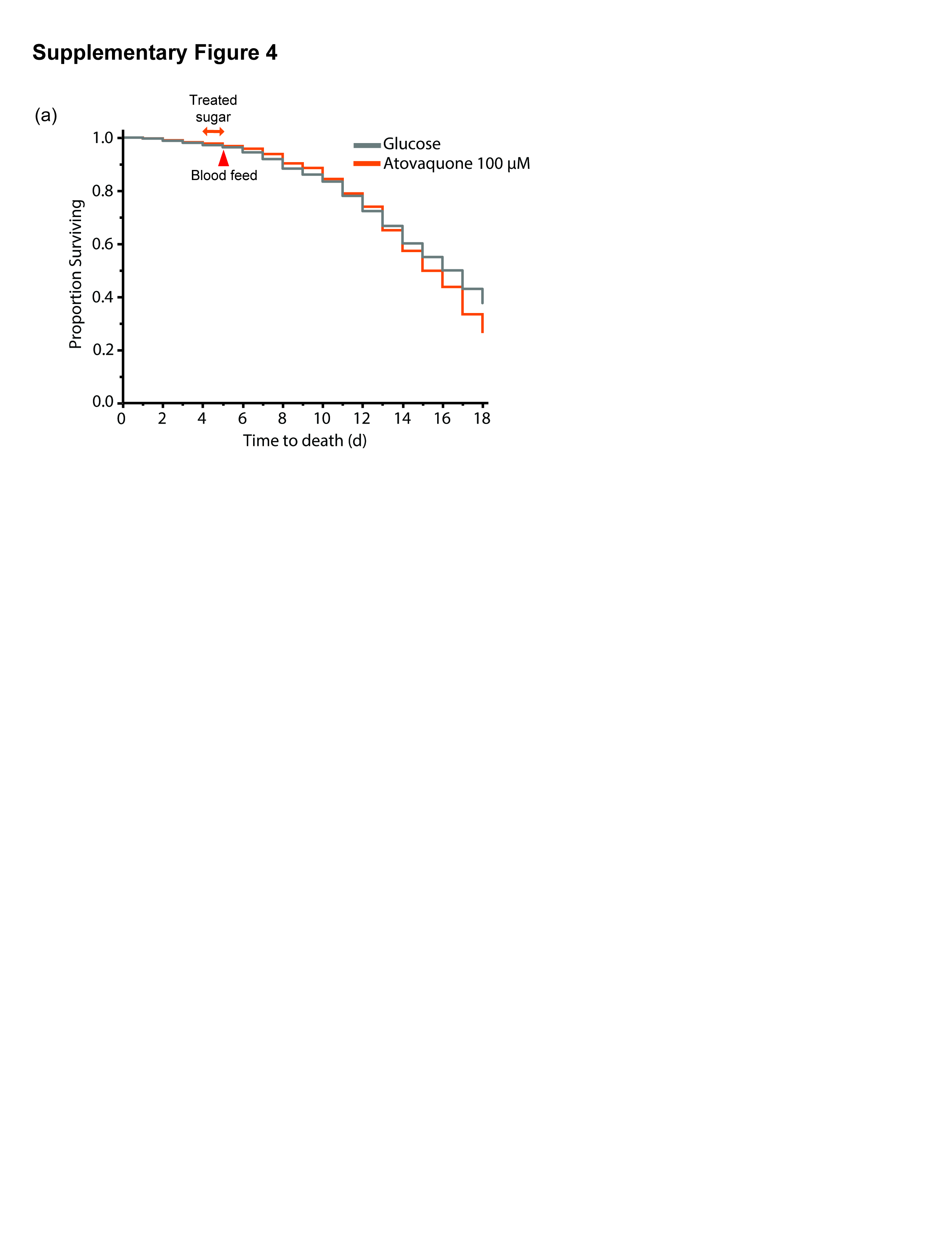

Supplement: S4 Fig — (a) Survival prior to- and following 24 h access to 100 μM/10% w/v Glucose/0.5 v/v/ DMSO (4 d post emergence, orange arrow), followed by P. falciparum (donor blood) infection (5 d post emergence, red arrow). Ingestion of ATQ/glucose had no impact on the survival of AcVK5 mosquitoes relative to a control group provided with 10% w/v glucose/0.5% v/v DMSO (Log-Rank Survival, n = 1374, df = 1, Χ2 = 1.3795, p = 0.2402). (TIF) [file ppat.1010609.s004.tif]

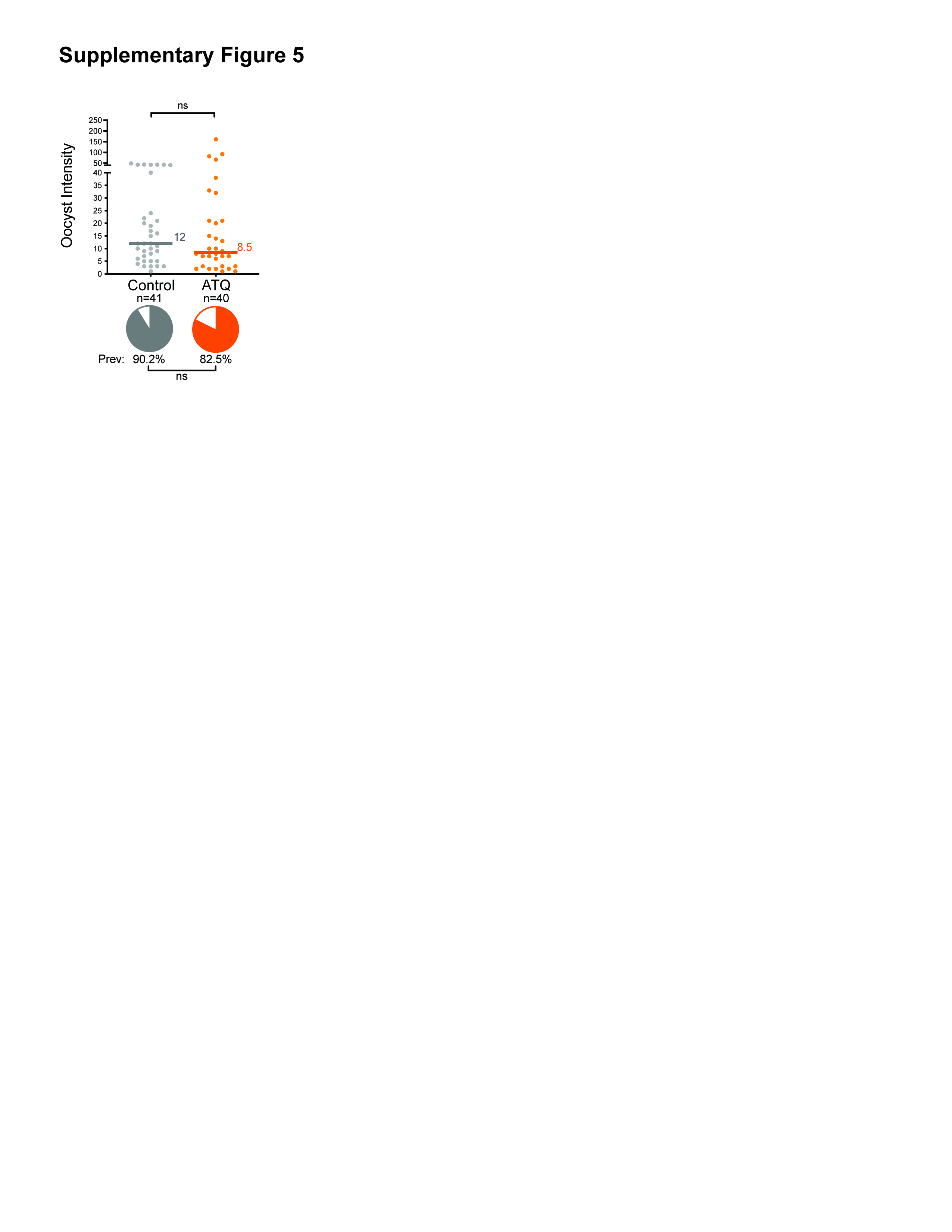

Supplement: S5 Fig — There was no difference relative to controls in either intensity (n = 68, df = 1, U = 495.5, p = 0.3257) or prevalence (n = 81, df = 1, Χ2 = 2.441, p = 0.1182) of oocysts at 10 d pIBM in females with continued access to 100 μM ATQ/0.5% DMSO/10% w/v glucose from 2 d pIBM—14 d pIBM. Median lines and values are indicated, “n” indicates the number of independent samples. To isolate Oocyst Prevalence and Oocyst Intensity, midgut samples with zero oocysts have been excluded from intensity analysis. Statistical significance is indicated where relevant as follows: ns = not significant, * = p<0.05, ** = p<0.01, *** = p<0.001, **** = p<0.0001. (TIF) [file ppat.1010609.s005.tif]
